# Convolutional low-rank adaptation for efficient semantic segmentation in vision transformers

**DOI:** 10.1038/s41598-026-57622-y

**Published:** 2026-06-13

**Authors:** Srihari Srinivasan, Morvin Prajapati, Ananthakrishna Thalengala, Deepu Raveendran, Chevuru Sai Jaswanth

**Affiliations:** 1 Manipal Institute of Technology, Manipal Academy of Higher Education, Manipal, India; 2 Samsung Research Institute Bangalore (SRIB), Bangalore, India

**Keywords:** Vision transformer, Depth anything V2, Convolution low-rank adaptation, Parameter-efficient fine tuning, Human semantic segmentation, industry, innovation and infrastructure (SDG-9), Computational biology and bioinformatics, Engineering, Mathematics and computing

## Abstract

Vision Transformers (ViTs) have shown remarkable performance across various computer vision tasks, but their fine-tuning for dense prediction tasks such as semantic segmentation remains computationally intensive. This work proposes a novel dual-task architectural application of the LyCORIS Low-Rank Adaptation for Convolutions (LyCORIS LoCon) framework, which introduces learnable low-rank convolutional modules into pre-trained ViTs. This method is applied to Depth Anything V2 (DAV2), augmenting its decoder to support dual-task outputs; monocular depth estimation and binary human semantic segmentation, without disrupting its original capabilities. By injecting only 150K trainable parameters, this approach significantly reduces the adaptation cost while achieving segmentation performance comparable to state-of-the-art models like SAM, MaskFormer, and SegFormer. Extensive experiments on filtered COCO^1^ and ImageNet subsets show that Conv-LoRA enhances task-specific learning with minimal computational overhead. The method achieves an mAP of 89.69% and an mIoU of 79.17% for human segmentation, performing competitively alongside state-of-the-art models like Mask2Former, while preserving the depth prediction accuracy of the base model.

## Introduction

The rise of large-scale pre-trained models in computer vision has paved the way for significant advancements across various tasks. However, adapting such models to new domains or tasks often involves resource-intensive fine-tuning, which is impractical in resource-constrained environments.

This work addresses the challenge of efficient adaptation by employing LyCORIS Low-Rank Adaptation for Convolutions framework (LyCORIS LoCON), a low-rank adaptation technique, to fine-tune the Depth Anything V2 (DAV2)^1,2^ model for binary human segmentation. With only 150k additional trainable parameters, LoCON achieves task-specific updates while preserving the pre-trained parameters of DAV2.

Fine-tuning large-scale vision models requires substantial computational resources and memory, limiting their accessibility in resource-constrained settings. This study aims to show that parameter efficient fine-tuning Convolution layers, although they are lighter in parameters than Attention layers of Transformers, are effective in fine-tuning large vision models to completely different tasks while using a fraction of the parameters than normally required.

Recent works have also explored novel strategies to adapt or repurpose vision transformers for dense prediction tasks without full fine-tuning^3^. In January 2024, a study introduced non-parametric operators–namely, token clustering and token construction layers–to expedite large-scale vision transformers by reducing and later restoring token resolution, effectively enabling dense prediction without altering model weights^4^. Complementing this, Tobias^5^ revealed that convolutional neural networks inherently exhibit object-centric inductive biases, even when untrained, suggesting that CNN architectures remain competitive for semantic localization tasks.

Around the same time, Medical SAM^6^ demonstrated the viability of lightweight adapters to specialize foundation models like SAM for medical applications, specifically enhancing glioma segmentation in brain MRIs. These advancements collectively emphasize a growing shift toward minimal-parameter, architecture-aware tuning strategies–supporting investigation into convolutional low-rank adaptation for high-capacity models like DAV2.

In the evolving landscape of vision model training, recent approaches have combined architectural innovations with powerful pretraining paradigms. Contrastive Masked Autoencoders (CMAE)^7^ merged contrastive learning with masked image modeling using a dual-branch setup–an asymmetric encoder-decoder as the online branch and a momentum-updated encoder as the target branch. This hybrid design enabled representations that are both instance-discriminative and perceptually localized, achieving state-of-the-art results across classification, segmentation, and detection benchmarks. In May 2024, the Quadrangle Attention Vision Transformer (QFormer)^8^ addressed the rigidity of hand-crafted window designs by introducing a learnable quadrangle regression module. This module dynamically adapts attention windows to object geometry, improving segmentation by capturing spatial context more effectively. These developments further reinforce the need for adaptive and efficient architectural mechanisms–both in token representation and attention modeling–motivating the use of convolution-based low-rank adapters to tune high-capacity transformer decoders like DAV2.

Efforts to reconcile the strengths of convolutional networks with transformer architectures have gained traction, particularly in addressing the limitations of multi-head self-attention (MSA). In May 2024, single path vision transformer (SPViT)^9^ introduced a single-path pruning framework that compresses pre-trained vision transformers by integrating convolutional locality into the attention mechanism. Through a novel weight-sharing scheme between MSA and convolutional layers, SPViT selects parameter subsets that retain global context while injecting spatial bias, effectively reducing compute demands and enhancing local pattern recognition. In parallel, a convolutional to transformer (Conv2Former)^10^ proposed a streamlined convolutional network architecture inspired by transformer design principles. By replacing complex attention modules with convolutional modulation operations and scaling kernel sizes up to 21*×*21, Conv2Former achieved consistent gains in visual recognition tasks while preserving architectural simplicity. These works highlight the efficiency and expressiveness of convolutional alternatives to self-attention, further validating the exploration of convolutional low-rank adaptation for high-capacity vision models like DAV2.

The recent wave of transformer enhancements has also focused on boosting spatial adaptability and resolution awareness for dense prediction tasks. The cross CNN transformer^11^, introduced in May 2024, extended its predecessor by integrating a cross-scale embedding layer (CEL) and long-short distance attention (LSDA) to better fuse multi-scale features while reducing computational overhead. The addition of progressive group sizing and amplitude cooling layers further stabilized attention scaling, enabling the model to perform exceptionally with advanced segmentation heads such as the unified perceptual parsing network (UPerNet).

Complementing this, the high input resolution input vision transformer (HIRI-ViT)^12^ tackled the challenge of maintaining high-resolution input fidelity by designing a hybrid 5-stage backbone with parallel CNN branches–one that processes high-resolution input with minimal computation. In addition it processes down-sampled input with deeper convolutions. This design achieved the best mean intersection over union (mIoU) within its category, showcasing how hierarchical, resolution-aware architectures can maintain performance under tight resource budgets. Together, these advances underscore a clear trend toward architectural efficiency and task adaptability–core motivations behind this proposal to fine-tune DAV2 through lightweight, convolutional low-rank adaptation for binary human segmentation.

Fine-tuning large-scale vision models requires substantial computational resources and memory, limiting their accessibility in resource-constrained settings. While previous approaches often require full network fine-tuning or entirely new architectures, our method addresses these limitations by introducing Convolutional Low-Rank Adaptation (Conv-LoRA). By injecting only 150K trainable parameters, the proposed approach efficiently adapts the Depth Anything v2 (DAV2) model for dual-task outputs–monocular depth estimation and binary human semantic segmentation–without disrupting its original capabilities.

Beyond structural modifications, the conceptual novelty of this work lies in introducing a ’zero-interference multi-task adaptation’ paradigm. Traditionally, adapting a frozen foundation model for multi-task outputs requires adding heavy, task-specific heads that risk catastrophic forgetting or task interference. The proposed study demonstrates that highly targeted, hierarchical parameter injections within a frozen continuous-prediction decoder can seamlessly bifurcate semantic knowledge (segmentation) from geometric knowledge (depth) without degrading the original zero-shot capabilities of the base model. The core contributions of the present study involves a tailored Conv-LoRA dual-path architecture that effectively adapts the DAV2 DPT decoder for dual-task prediction. A hierarchical bilinear up-sampling combined with independent sigmoid-normalized paths prevents destructive interference between continuous depth prediction and binary segmentation. A competitive multi-task performance has been acheived with only 150K trainable parameters, significantly lowering the barrier for deployment in resource-constrained settings.

## Related work

The rapid growth of pre-trained models has driven the need for parameter-efficient fine-tuning methods to address their computational and memory demands. Low-Rank Adaptation (LoRA) achieves this by injecting trainable low-rank matrices into pre-trained Transformer layers, significantly reducing the number of trainable parameters and GPU memory requirements^13^. Building on this foundation, Weight-Decomposed Low-Rank Adaptation (DoRA) refines the process by decomposing pre-trained weights into magnitude and direction, enabling more efficient updates while maintaining training stability^14^.

Various extensions of LoRA have been developed to further optimize memory usage and parameter allocation. To tackle memory constraints in large models, quantized low rank adaptation (QLoRA) introduces 4-bit quantization and memory-efficient innovations, allowing fine-tuning of massive models on hardware with limited resources^15^. Extending LoRA’s efficiency, molecular ranking adaptation (MoRA) incorporates high-rank updates with square matrices, enhancing the model’s learning capacity for memory-intensive tasks without additional deployment complexity^16^.

Recognizing the need for adaptive parameter allocation, adaptive low-rank adaptation (AdaLoRA) dynamically assigns parameter budgets based on importance scores, ensuring efficient fine-tuning while retaining task performance across various natural language processing (NLP) tasks^17^. While LoRA-based methods have primarily focused on NLP, the Vision Transformer (ViT) demonstrates the efficacy of pure Transformer architectures for computer vision, outperforming traditional CNNs by applying attention mechanisms directly to image patches^18^. In the computer vision domain, Transformer-based models and their task-specific adaptations have demonstrated state-of-the-art performance. Expanding on the potential of Transformer-based models in vision, distillation of knowledge with no labels verion-2 (DINOv2) employs self-supervised learning on curated datasets, scaling model and data size to establish robust, general-purpose visual features^19^. Complementing this, the Segment Anything Model (SAM) introduces a promptable segmentation framework, efficiently combining flexible encoding and decoding components for real-time, scalable segmentation tasks^20^.

The pursuit of more efficient and generalizable vision backbones has been a major research focus since 2022. In April 2022, simplified masked image modeling (MIM)^21^ introduced and is an effective strategy that used random masking of moderately large patches and predicted raw RGB values through direct regression. Remarkably, its lightweight prediction head, comparable to a linear layer, outperformed heavier alternatives in both classification and semantic segmentation tasks. The results have shown +0.6% gain on annotated scene centric image data set of size 20000 (ADE20K) with vision transformer base model (ViT-B). Building on these ideas, Convolutional Masked autoencoders (ConvMAE)^22^ further demonstrated that hybrid convolution-transformer backbones benefit from the MIM paradigm, leveraging hierarchical representations to bridge the transfer gap between pre-trained and downstream networks. This approach effectively fused the strengths of convolutional locality and transformer-based global reasoning.

In August 2022, the region attention based shifted window (RA-Swin) strategy^23^ has been explored for depth estimation by proposing a Transformer-based shifted window encoder-decoder pipeline. Through shifted window attention and a spatially adaptive decoder built on refinement network (RefineNet), it achieved fine-grained depth predictions with fewer parameters by integrating multi-scale features efficiently. These works collectively laid the groundwork for lightweight yet powerful adaptation strategies that inspire the use of low-rank convolutional fine-tuning in large-scale models like DAV2.

To address SAM’s limitations in specialized domains, Convolution Meets LoRA (Conv-LoRA) integrates convolutional biases into LoRA, enhancing SAM’s adaptability for domain-specific applications like medical imaging and remote sensing^24^. Building on this, Depth Anything V1 emphasizes large-scale dataset collection and auxiliary supervision, achieving robust monocular depth estimation across diverse environments^25^.

Recent studies have also highlighted the importance of efficient multi-modal feature fusion in Transformer networks. Works such as TwinsTNet^26^ for broad-view bimodal salient object detection, Deep Fourier-Embedded Networks^27^ for RGB-Thermal detection, and Efficient Fourier Filtering Networks^28^ for unaligned bimodal detection showcase advanced strategies for managing dual-task feature representations. These methods inform our dual-task Conv-LoRA architecture, emphasizing the need for shared representations that can be effectively split without task degradation.

Furthermore, recent studies such as Umirzakova et al.^29^ highlight the necessity of iterative contextual and adaptive strategies for the enhanced monocular depth estimation, underscoring the demand for robust generalization across domains. Recent advancements in monocular depth estimation and convolutional adaptations lay the groundwork for our proposed method. Improving upon its predecessor, DAV2 introduces synthetic training data and advanced teacher-student learning strategies, resulting in scalable models with superior depth estimation capabilities^2^. Finally, LoRA beyond conventional methods, other rank adaptation implementations for stable diffusion (LyCORIS) incorporates convolutional adaptations with low-rank methods, fine-tuning the final decoder of DAV2 to optimize segmentation performance in domain-specific tasks^30^.

In addition to weight adaptation, prompt tuning and instruction tuning have emerged as efficient alternatives for downstream task adaptation. Works such as effective and efficient visual prompt tuning (E2vpt)^31^ and capsule prompt tuning^32^ with a single vector have demonstrated the efficacy of prompt tuning for visual understanding, significantly reducing parameter overhead. Similarly, representation-focused methods like ’Re-imagining multimodal instruction tuning’^33^ have advanced instance tuning for dense downstream tasks like segmentation. While these methods optimize input prompts or instance-level instructions, our Conv-LoRA approach fundamentally differs by directly injecting low-rank convolutional weight adaptations into the decoder’s hierarchical structure.

## Methodology

### Preliminaries

#### Depth anything V2

Depth Anything V2 represents a significant advancement in monocular depth estimation by leveraging a multi-stage training pipeline^2^. The methodology in depth anything v2 begins with training a large-scale teacher model based on the DINOv2-Giant backbone. This model is trained exclusively on a dataset of approximately 595,000 synthetic images to establish a robust understanding of depth estimation in a controlled synthetic environment^19^.

The trained teacher model is then utilized to generate high-quality pseudo-depth labels for over 62 million unlabeled real-world images. This step is crucial to bridge the domain gap between synthetic and real-world datasets and enhance the model’s generalization capability.^25^

The final stage involves training student models on the pseudo-labeled real-world dataset. This step ensures that the model learns finer depth estimation features and adapts effectively to real-world scenarios.

The DAV2 model employs a robust DPT head decoder composed of ResNet-based convolutional layers^34^. This decoder is designed to effectively capture multi-scale features and refine depth predictions by leveraging residual connections^34^. ResNet convolutions in the decoder provide a strong capability for spatial feature integration, enhancing depth estimation accuracy^35^.

Depth Anything V2 (DAV2) has been selected as the base model due to its robust DPT head decoder composed of ResNet-based convolutional layers. This decoder architecture is exceptionally well-suited for our proposed method, as the ResNet convolutions provide a strong capability for spatial feature integration, making them an ideal foundation for injecting convolutional low-rank adaptations (LoCON).

#### Low-rank adaptation (LoRA)

Low-Rank Adaptation (LoRA) builds on the premise that fine-tuning updates exhibit a low intrinsic rank, enabling efficient adaptation of pre-trained models^13^. LoRA introduces a pair of low-rank matrices to approximate weight updates, significantly reducing the number of trainable parameters while maintaining the expressiveness of the model. LoRA introduces trainable low-rank matrices to adapt large models efficiently by approximating weight updates.

Given a pre-trained weight matrix $W_0 \in \mathbb {R}^{d \times k}$, LoRA models the weight update $\Delta W \in \mathbb {R}^{d \times k}$ as the product of two low-rank matrices, $B \in \mathbb {R}^{d \times r}$ and $A \in \mathbb {R}^{r \times k}$, where $r \ll \min (d, k)$. The adapted weight *W'* is computed as:

1 $$\begin{aligned} W' = W_0 + BA \end{aligned}$$

where $W_0$ remains frozen during training, and only the parameters of *B* and *A* are updated. The initialization ensures *B* is set to zero and *A* is initialized using the Kaiming distribution, making *Δ W* initially zero. This setup allows the model to leverage pre-trained knowledge while introducing minimal overhead.

#### LyCORIS LoCon: low-rank adaptation for convolutions

The LyCORIS LoCon framework extends the principles of Low-Rank Adaptation (LoRA) to convolutional layers, enabling efficient fine-tuning of pre-trained convolutional neural networks (CNNs)^36^.

For a convolutional weight tensor $\mathcal {W}_0 \in \mathbb {R}^{C_{\text {out}} \times C_{\text {in}} \times H \times W}$, LoCON introduces two trainable low-rank matrices, $\mathcal {B} \in \mathbb {R}^{r \times C_{\text {in}} \times H \times W}$ and $\mathcal {A} \in \mathbb {R}^{C_{\text {out}} \times r \times 1 \times 1}$, where $r \ll \min (C_{\text {out}}, C_{\text {in}})$. The weight update is expressed as:

2 $$\begin{aligned} \mathcal {W}' = \mathcal {W}_0 + \mathcal {A} \cdot \mathcal {B} \end{aligned}$$

where *·* denotes matrix multiplication. Here, $\mathcal {B}$ reduces the input dimensionality to a smaller rank *r*, and $\mathcal {A}$ restores the rank to match the output channels.

By freezing $\mathcal {W}_0$ during training and updating only the low-rank matrices, LoCON reduces the number of trainable parameters while retaining the expressive power of the original model.

To ensure that the newly learned semantic features do not redundantly overlap with the frozen geometric features, the orthogonality constraint on the low-rank matrices during training have been introduced. The regularization term is defined, which enforces sparsity and encourages the adapter to learn strictly complementary task-specific representations.

### Modification to decoder architecture

#### Integration of LoCON adapter layers into DPT decoder convolutions

The integration of Low-Rank Adaptation for Convolutions (LoCON) into the DAV2 architecture enhances the fine-tuning efficiency of the model by introducing lightweight and adaptive updates to convolutional layers. These LoCON modules are embedded into the DPT head’s convolutional layers, enabling task-specific adaptation while preserving the pre-trained knowledge of the base model.

During the training phase, the base DAV2 convolutional weights are strictly frozen to preserve pre-trained geometric knowledge. Only the injected low-rank matrices receive gradient updates through the combined loss function. During the inference phase, the forward pass utilizes the shared feature representations up to the divergence node, at which point the processing splits into two distinct paths: (1) The original depth estimate, which is computed exclusively using the frozen weights. (2) The segmentation mask, which is computed using the LoCON-enhanced weights. This node-level divergence ensures the segmentation learning does not interfere with the base model’s depth prediction. The primary advantage of integrating LoCON directly into the DPT decoder convolutions is that it preserves the base model’s pre-trained global reasoning capabilities while enabling highly targeted, task-specific spatial adaptation for segmentation.

LoCON decomposes the weight updates of each convolutional layer into two low-rank matrices. During inference, they are multiplied and the result is added to the existing pre-trained convolutional weight matrices.

**Fig. 1:**
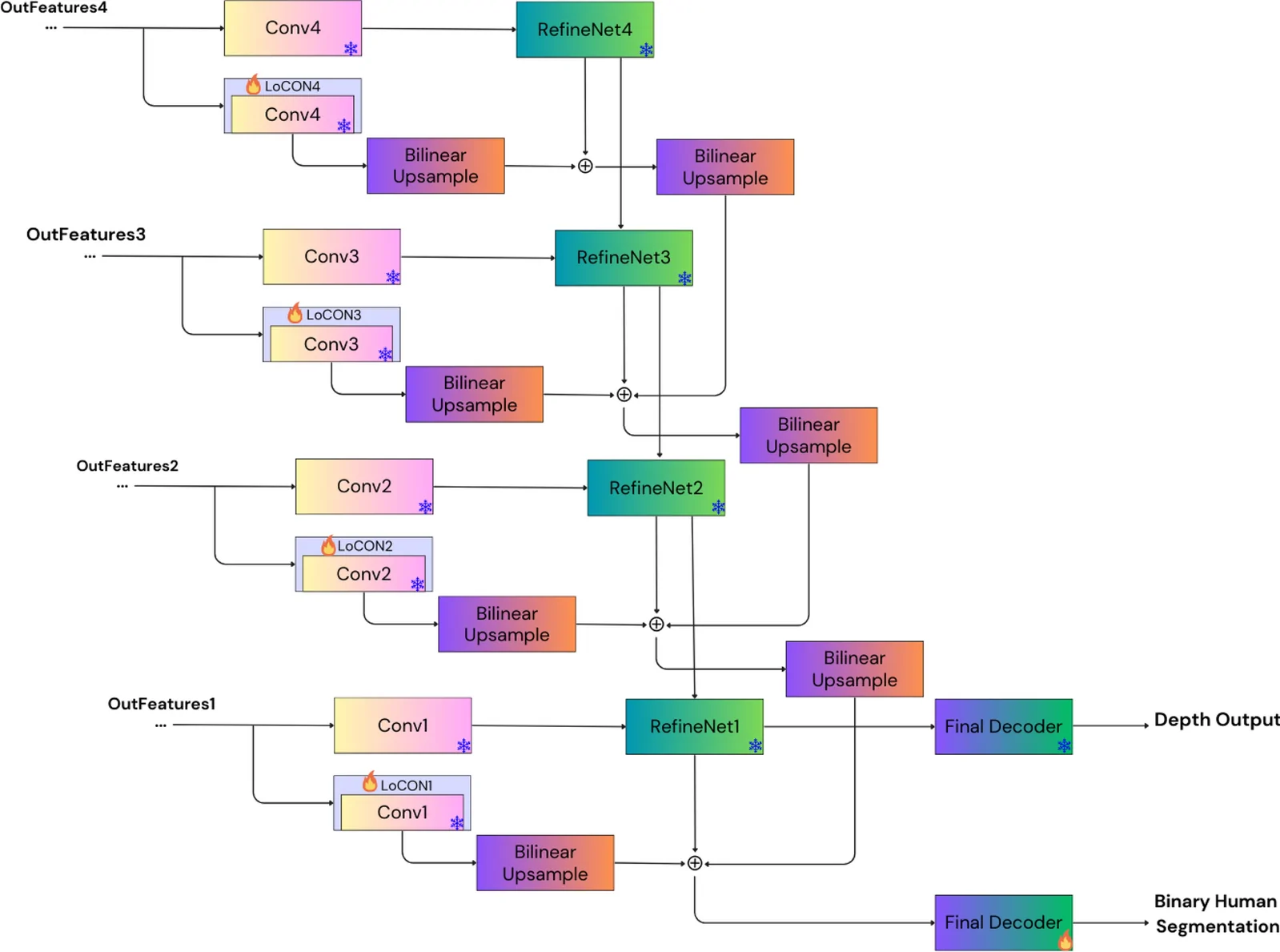
Modified DPT head decoder with dual-path outputs.

**Fig. 2:**
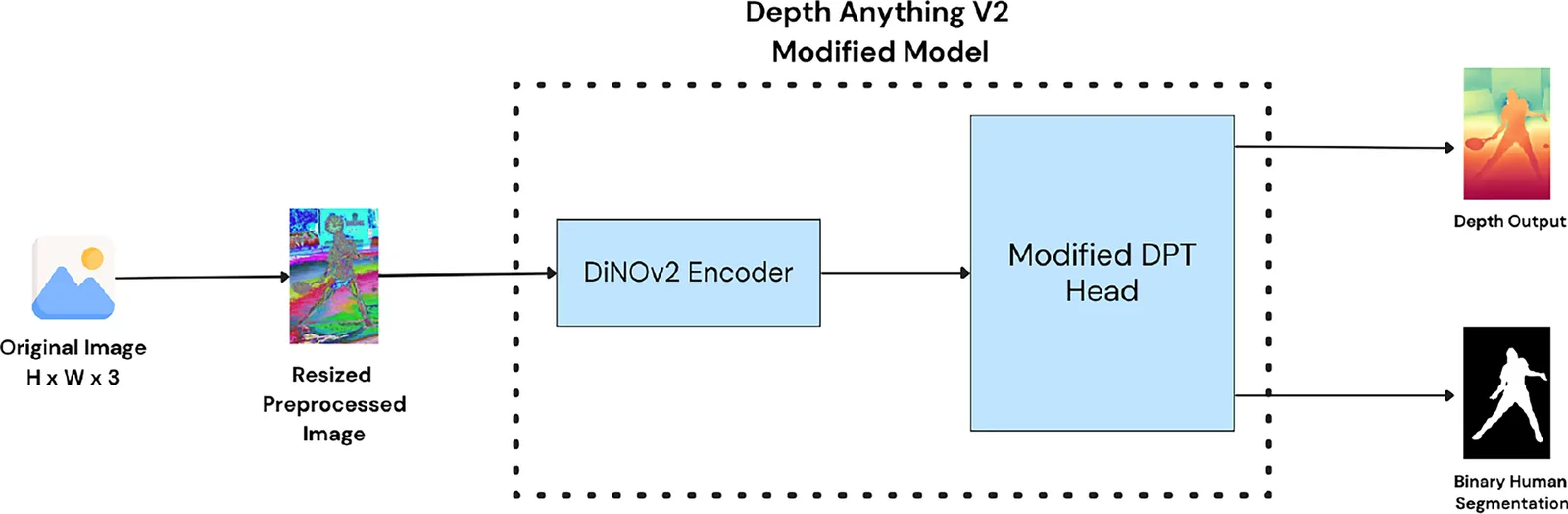
Overview of the modified DAV2 architecture.

**Fig. 3:**
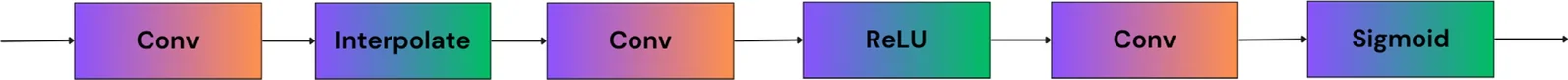
Final decoder architecture for depth and segmentation outputs.

#### Fusion of LoCON output with depth features using up-sampling

This architecture incorporates LoCON-enhanced convolutional outputs into the feature refinement process by merging these outputs with depth features in a hierarchical manner. This process ensures efficient feature fusion across multiple scales, contributing to robust depth prediction and segmentation results. The advantage of this hierarchical fusion through bilinear up-sampling is that it allows the model to progressively integrate finer details, providing the robust context-awareness essential for dense prediction tasks. The rationale behind this dual-path design is to facilitate task-specific migration by allowing the model to share feature extraction computations through the initial hierarchy. By splitting into two independent paths at the final layers; each normalized through independent sigmoid functions. The architecture prevents destructive interference between the continuous depth prediction and the binary segmentation task, ensuring stable optimization for both outputs.

LoCON outputs from convolutional layers are first up-sampled to match the spatial resolution of the refinement path output by the Residual Network of CNNs. The up-sampled LoCON features are merged with the intermediate depth features produced by the decoder using element-wise addition. This hierarchical fusion is performed at multiple levels, progressively integrating finer details and context-aware information as the features are propagated through the decoder. The merged features F’ is modelled as:

3 $$\begin{aligned} F' = U(F_{\text {LoCON}}) + F_{\text {Decoder}} \end{aligned}$$

where $F_{\text {LoCON}}$ represents the output features from LoCON-enhanced convolutions, $F_{\text {Decoder}}$ denotes the intermediate features from the decoder, and *U(· )* is the bilinear upsampling operation. The merged features *F'* are then passed to subsequent refinement layers for further processing.

The modified decoder incorporates LoCON modules for low-rank updates to convolutional layers, enabling segmentation alongside depth estimation. Figure 1 illustrates the architecture of the modified DPT head. Trainable LoCON Layers wrap around the frozen Convolution weights, passing the output to Bilinear Upsampling to match the dimensions of the RefineNet of DPT Head. The output is merged with the other features and the two outputs are passed through identical final decoder architectures

#### Dual-path decoder architecture

The proposed architecture builds upon DAV2, retaining its DINOv2 encoder and depth head while introducing modifications in the decoder to produce dual outputs: depth maps and binary human segmentation masks. The overall architecture is shown in Figure 2.

The original image is preprocessed identical to DAV2 and passed into the unmodified DiNOv2 encoder. The output is passed down the pipeline into the modified DPT head which provides the depth map of the base model and the human semantic segmentation.The model incorporates a dual-path decoder architecture, designed to simultaneously produce depth and segmentation outputs from a shared feature representation. The decoder operates hierarchically, progressively refining features from the encoder through a series of convolutional and up-sampling layers. These refined features are then split into two independent paths namely depth path and segmentation path. In depth path, there will be a continuous output with a single-channel prediction representing the depth values normalized using a sigmoid activation function. Whereas in the segmentation path produces a binary mask, highlighting regions of interest (e.g., human segmentation), also normalized using a sigmoid activation function.

The first path carries the convolution operation carried out through the original weight matrices to produce the unmodified depth estimate and the second path holds the convolution operation carried through the updated weight matrices which will become the parallelly obtained binary human segmentation mask.

The final layers of the decoder for both the depth and segmentation paths are distinct instances of the same architectural design, as shown in Figure 3. The decoder architecture of DPT head is retained but the weights are trainable for the human semantic segmentation output.

Unlike standard LoCon applications that merely adapt single-task classification or detection pipelines, the proposed tailored design wraps the frozen convolution weights while utilizing hierarchical bilinear up-sampling to merge features. This allows the model to share a unified feature representation that smoothly diverges into independent, sigmoid-normalized depth and segmentation paths. This shared design ensures efficiency while preserving task-specific optimization, which is adapted from the original DAV2 decoder output layer. The process for generating each output can be expressed as:

4 $$\begin{aligned} O = \sigma (\text {Conv}(\text {Interpolate}(\text {Conv}(\mathcal {F})))) \end{aligned}$$

where $\mathcal {F} \in \mathbb {R}^{C_{\text {in}} \times H \times W}$ represents the shared input feature map. The first *Conv*(.) reduces the channel dimension to an intermediate size, *Interpolate*(.) performs bilinear up-sampling to the target spatial resolution, and the final *Conv*(.) projects the features to $C_{out} =1$. Finally, *σ (.)* applies the sigmoid activation function to yield either the normalized depth map or the binary segmentation mask depending on the path.

The final layers of the decoder for both the depth and segmentation paths are distinct instances of the same architectural design, which is adapted from the original DAV2 decoder output layer. The design initially optimized for depth prediction proves effective for segmentation tasks as well, maintaining architectural efficiency.

## Experiments

### Datasets

For training and evaluation, carefully curated subsets of the COCO and ImageNet datasets were employed. These subsets were filtered to contain images with a significant presence of humans, determined based on pixel-wise thresholds for human occupancy. This selection ensures that the model is exposed to challenging scenarios while focusing on the primary task of binary human segmentation. COCO provides a diverse set of real-world contexts with varied poses and occlusions, while ImageNet adds further variability in terms of lighting, resolution, and background clutter. These choices enable a comprehensive assessment of the model’s generalization ability across both controlled and unconstrained environments.

The training dataset comprised 15,000 images from the filtered COCO subset and 8,000 images from ImageNet, totaling 23,000 training samples. The validation set contained 2,500 images (1,500 from COCO and 1,000 from ImageNet), while the test set included 3,000 images (2,000 from COCO and 1,000 from ImageNet). All images were resized to 518*×*518 pixels to match the input requirements of the DAV2 architecture.

### Training configuration and experimental setup

The experimental setup utilized PyTorch framework with mixed precision training on NVIDIA A100 GPUs. The base DAV2 model weights were frozen, with only the LoCON adapter parameters being trainable, resulting in 150,000 additional parameters. Training was conducted for 50 epochs using the AdamW^37^ optimizer with a learning rate of $10^-4$, weight decay of $10^-2$, and a cosine annealing learning rate scheduler.

The batch size was set to 16 due to GPU memory constraints, and gradient accumulation over 4 steps was employed to achieve an effective batch size of 64. Data augmentation techniques included random horizontal flipping, color jittering, and random rotation within ±10 degrees. The total loss function combined binary cross-entropy loss for segmentation, L1 loss for depth preservation, and the orthogonality penalty , weighted at 0.7, 0.3, and 0.01 respectively.

### Evaluation metrics

To quantitatively evaluate the performance of the proposed method, two widely accepted metrics in semantic segmentation tasks were employed: mean Average Precision (mAP) and mean Intersection over Union (mIoU). These metrics provide a balanced view of both precision and overlap with the ground truth segmentation masks.

The mean Average Precision is computed as:

5 $$\begin{aligned} \text {mAP} = \frac{1}{N} \sum _{i=1}^{N} \text {AP}_i \end{aligned}$$

where $\text {AP}_i$ is the Average Precision for class *i*, calculated as:

6 $$\begin{aligned} \text {AP}_i = \int _{0}^{1} P(R) \, dR \end{aligned}$$

with *P*(*R*) representing the precision-recall curve.

The mean Intersection over Union is defined as:

7 $$\begin{aligned} \text {mIoU} = \frac{1}{N} \sum _{i=1}^{N} \frac{TP_i}{TP_i + FP_i + FN_i} \end{aligned}$$

where $TP_i$, $FP_i$, and $FN_i$ represent true positives, false positives, and false negatives for class *i*, respectively.

### Baseline models

The model was benchmarked against three strong baselines–SAM, Mask2Former, and SegFormer–all of which represent state-of-the-art architectures optimized for segmentation. In addition to full-network architectures (SAM, Mask2Former, SegFormer), model is benchmarked against leading parameter-efficient fine-tuning (PEFT) baselines, including LoRA-ViT, visual Adapter-based methods, and standard Conv-LoRA, all applied to the same DAV2 backbone. These models serve as rigorous reference points for assessing the effectiveness of the lightweight fine-tuning strategy using LoCON. All baseline models were evaluated using their official implementations and pre-trained weights, with identical test datasets to ensure fair comparison.

## Results and discussion

### Quantitative results

The proposed model achieved performance metrics that are competitive with the state-of-the-art, all while operating with significantly reduced computational overhead. Table 1 presents a comparative overview of mean AP and mean IoU across all tested models.

On the filtered COCO dataset with 1751 high quality human-centric images, the proposed approach achieved a mean Average Precision (mAP) of 0.8969 and a mean Intersection over Union (mIoU) of 0.7917, positioning it just behind Mask2Former, while outperforming both SAM and SegFormer.

For the ImageNet-based experiment, ground truth segmentation masks were generated using SAM on a filtered subset of 4566 images focused specifically on human-centric categories. Given that SAM was also used to generate the ground truth masks, its evaluation on this dataset serves more as a sanity check than a competitive benchmark. On this subset, the proposed method achieved an mAP of 0.8560 and an mIoU of 0.7683, again outperforming SegFormer and trailing just behind Mask2Former.

The proposed Conv-LoRA needs to be benchmarked against other standard Parameter-Efficient Fine-Tuning (PEFT) approaches such as LoRA, Adapter, visual prompt tuning (VPT), and weight-Decomposed Low-Rank Adaptation (DoRA). As shown in table 1, Conv-LoRA outperforms these alternatives in both mAP and mIoU, validating our convolution-specific low-rank design. These results highlight that even with only 150k additional parameters introduced via LoCON, the model is capable of delivering segmentation performance close to fully fine-tuned or dedicated segmentation models, thereby underscoring the efficiency of the approach.

**Table 1:** Comparison of MAP and MIOU on COCO and ImageNet datasets.

**Model**	**COCO**		**ImageNet**	
	**mAP**	**mIoU**	**mAP**	**mIoU**
Mask2Former	0.9076	0.8162	0.8677	0.7829
SAM	0.8877	0.7508	0.8406	0.7508
Segformer	0.8769	0.7489	0.8358	0.7491
**Proposed**	0.8969	0.7917	0.8560	0.7683

### Qualitative analysis

**Fig. 4:**
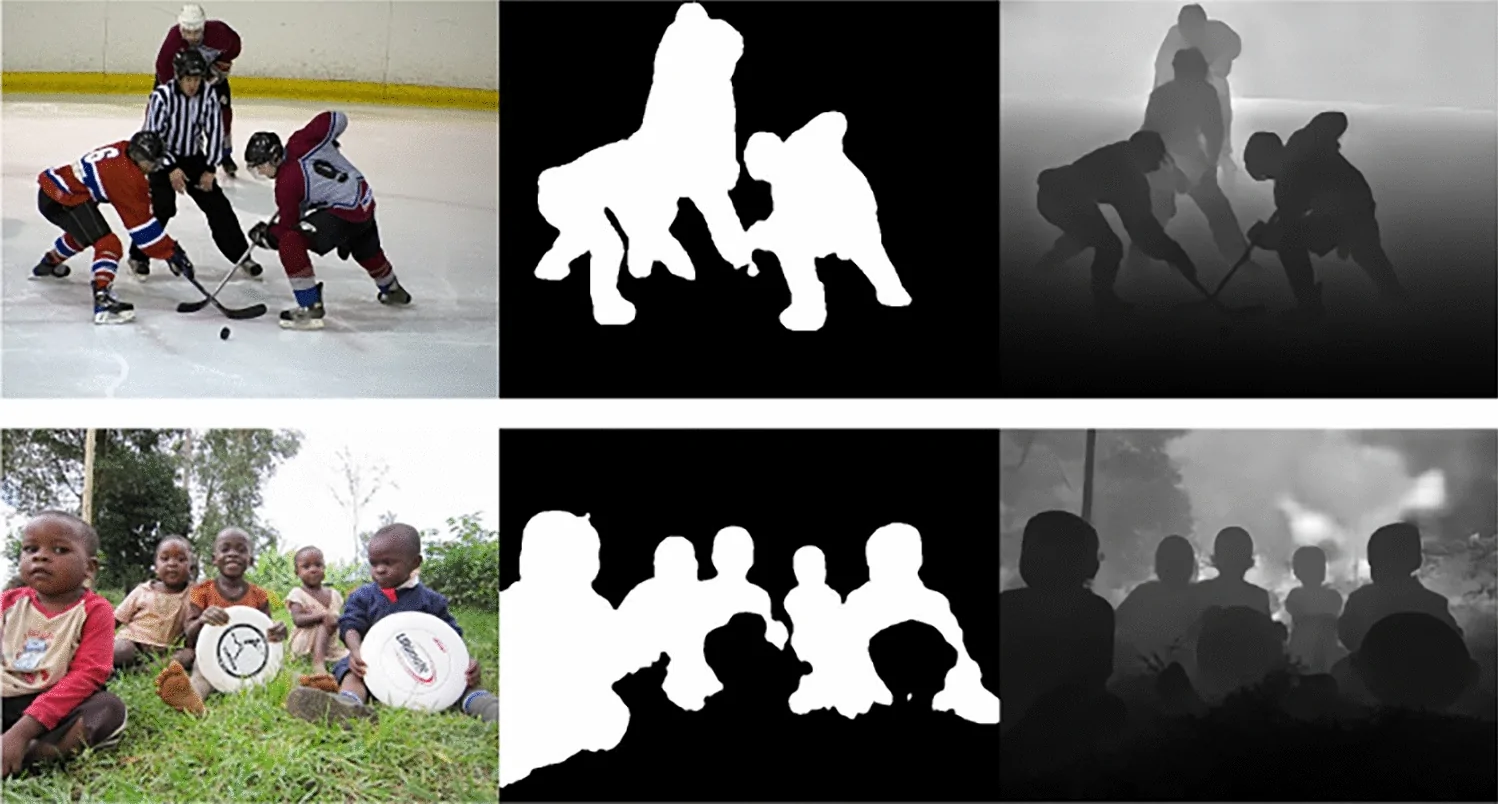
Qualitative results demonstrating model performance across diverse challenging scenarios: (Left) Original input images, (Center) Binary human segmentation masks with humans shown in white, (Right) Corresponding depth estimation outputs as grayscale depth maps.

Figure 4 shows the resulting images obtained from the proposed method. The figure demonstrates two representative examples showcasing the model’s performance across different challenging scenarios. Each row contains three images: the original input image, the predicted human segmentation mask (shown in white against black background), and the corresponding depth estimation output (shown as grayscale depth maps). The first example in figure  4 highlights the model’s effectiveness in segmenting multiple humans engaged in close physical interaction during a fast-paced activity. The second example showcases the model’s ability to handle complex multi-person scenarios with significant inter-person occlusions and varying poses. The outdoor group setting presents multiple individuals in close proximity, creating challenging segmentation conditions due to overlapping boundaries and diverse body orientations.

The qualitative results highlight the robustness and versatility of the segmentation approach across diverse challenging scenarios, including standard scenes, scale variations, multi-person environments, and unconventional human representations. The consistency in segmentation quality demonstrates that the model produces reliable human detection while maintaining the same high-quality depth outputs as Depth Anything V2. This consistency is achieved because the original model’s parameters remain unmodified, with only the LoCON adapters contributing to the segmentation task. The model’s ability to handle such diverse scenarios–from small-scale subjects to projected human images–indicates strong generalization capabilities and practical applicability across various real-world use cases.

### Performance analysis and discussion

The performance metrics in Table 1 demonstrate that the approach achieves competitive results compared to state-of-the-art segmentation models. The model’s mAP places it second only to Mask2Former, while surpassing both SAM and SegFormer. Similarly, the mIoU of outperforms SAM and SegFormer significantly.

What makes these results particularly noteworthy is the parameter efficiency of the approach. While achieving performance within 1.2% of Mask2Former’s mAP, the model requires only 150k additional trainable parameters–approximately 0.3% of what would be needed for full fine-tuning. This remarkable efficiency validates the core hypothesis that low-rank adaptation of convolutional layers can achieve near-SOTA performance with minimal computational overhead.

To demonstrate the robustness of the results, two key analyses were conducted. First, reproducibility was verified through multiple training runs with different random seeds, observing consistent performance with minimal variance in metrics. Second, testing on a separate validation set drawn from ImageNet showed only a minor performance decrease (less than 3% in both metrics), indicating strong generalization capability rather than dataset-specific optimization. This is due to the way fine-tuning is performed, where only a fraction of the parameters are being updated from the model.

These findings collectively demonstrate that the approach provides a practical and efficient solution for adapting large vision models to specific segmentation tasks without the computational burden of full fine-tuning, while maintaining performance comparable to specialized segmentation architectures.

**Fig. 5:**
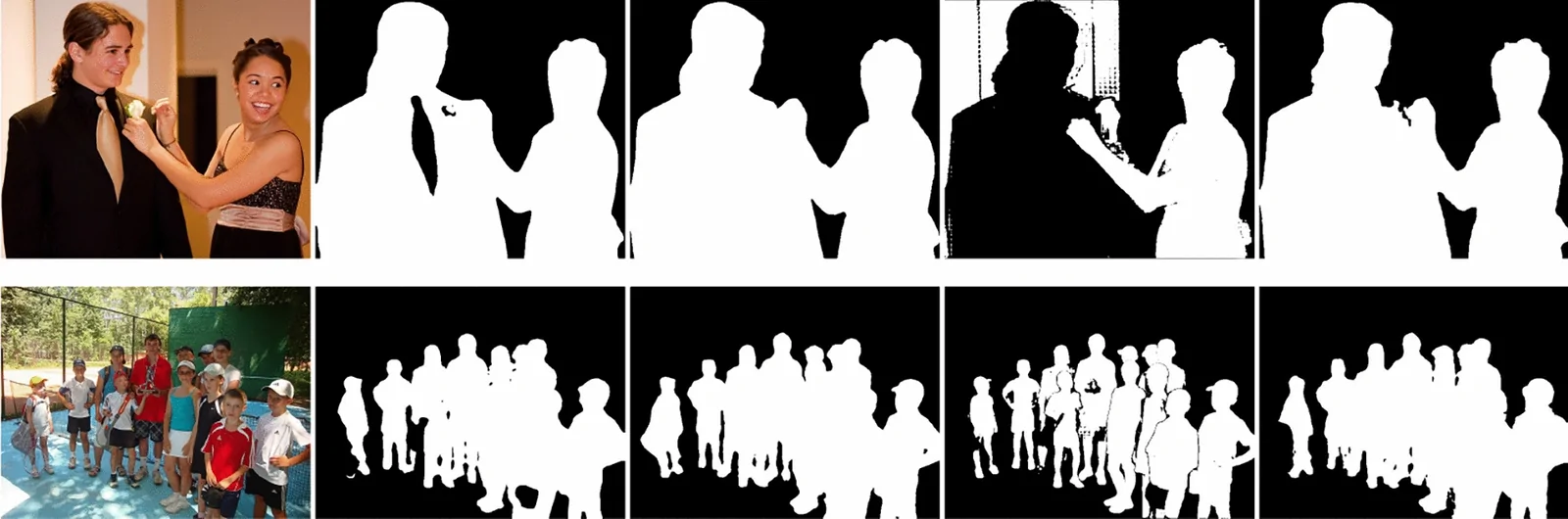
Comparison of segmentation outputs across various models. Original Image, Mask2Former Mask, Our Model Mask, SAM Mask, SegFormer Mask from Left to Right.

While Conv-LoRA demonstrates robust performance, it encounters limitations in scenes with extreme multi-person occlusions or severe lighting variations. In these edge cases, the shared hierarchical features occasionally struggle to differentiate deeply entangled limbs, resulting in minor segmentation bleed.

## Ablation study

In the first example of figure  5, our model demonstrates strong capability in accurately identifying all human figures and preserving their overall shape without missing essential regions or introducing noticeable segmentation bleed. The predicted masks are coherent and entity-consistent, though the model tends to produce softer, more heuristic boundaries rather than sharp edges. In contrast, Mask2Former often segments individual human components (e.g. accessories) as separate objects, leading to fragmented masks that fail to capture the subject as a unified whole. This behavior stems from its instance-level training bias, which is not ideal for holistic human segmentation tasks. SAM, while powerful and general-purpose, occasionally misclassifies or oversegments due to its exposure to a vast number of categories, causing confusion in human-centric scenes. SegFormer produces reasonably good masks but is prone to segmentation bleeding, particularly around overlapping or interacting body parts, reducing the clarity of person boundaries.

In the second example of figure  5, our model continues to exhibit strong recall in human segmentation by employing a more inclusive strategy around the human boundary. Although this occasionally results in auxiliary objects being incorporated into the segmentation mask, the approach ensures complete coverage of each individual without omitting body parts or creating fragmented masks. In contrast, Mask2Former suffers from clear bleeding issues, where human boundaries blend into the background or neighboring subjects, leading to inconsistent segmentation. By design, SAM segments each human instance individually with high pixel-level precision, but this instance-focused granularity often leads to structural inconsistencies or omissions, especially in crowded scenes. SegFormer struggles the most in this scenario, particularly in differentiating tightly packed individuals. Its prediction fails to maintain person-level separation, resulting in overly merged masks and poor localization performance.

To further validate the architectural choices, it is desirable to conduct quantitative ablation studies such as analysis of effect of rank (r), adapter placement study and LoCON versus standard LoRA camparative analysis. However, the results related to deeper ablation study has not been considered and explored.

## Conclusion and future work

This work has demonstrated the effectiveness of Low-Rank Adaptation for Convolutions (LoCON) in fine-tuning complex vision models for downstream tasks. By integrating only 150k trainable parameters into DAV2’s decoder, binary human segmentation has been achieved with performance competitive with specialized segmentation models, while maintaining the model’s original depth estimation capabilities.

The experimental results on COCO and ImageNet datasets validate that the proposed approach strikes an optimal balance between performance and efficiency. The model achieves an mAP of 0.8969 and mIoU of 0.7917, approaching state-of-the-art performance while using only a fraction of trainable parameters compared to full fine-tuning. This efficiency makes the approach particularly valuable for resource-constrained environments where computational overhead is a critical concern.

The success of LoCON adaptation for convolutional networks suggests that similar parameter-efficient techniques could be effective across a broader range of vision architectures beyond transformer-based models. This opens promising avenues for democratizing access to state-of-the-art computer vision capabilities in scenarios where computational resources are limited.

Future work could explore the application of this methodology to other vision tasks such as object detection and instance segmentation, as well as investigating the scalability of LoCON adaptations to even larger foundation models. Additionally, examining the combination of LoCON with complementary techniques like knowledge distillation could further enhance efficiency while preserving performance.

The ability to retain the original model’s depth estimation capabilities while seamlessly adding segmentation functionality demonstrates the flexibility and modularity of the LoCON approach. Rather than requiring the deployment of separate models for different tasks, this unified solution significantly reduces deployment complexity and resource consumption. As multimodal models become increasingly prevalent, this work presents a compelling case for lightweight task adaptation without sacrificing general-purpose utility – enabling broader adoption of foundation models in constrained settings such as mobile devices, robotics, and edge computing platforms.

Furthermore, the use of small, isolated subsets of trainable parameters for different downstream tasks makes it feasible to dynamically switch between capabilities–such as segmentation, depth estimation, or even pose detection–by simply loading the corresponding LoCON weights. This modularity facilitates real-time adaptation in multi-task systems without the need to reinitialize or fully retrain the core model, making it ideal for embedded AI applications, on-device personalization, and scalable multi-user deployments where each task or user may require a specialized behavior without incurring additional memory or compute overhead.

## Data Availability

The data sets used in the study are open resources available in the public domain. The URL link for the open-source dataset is https://cocodataset.org/#home, which does not require prior permission or consent.
